# The antioxidant property of CAPE depends on TRPV1 channel activation in microvascular endothelial cells

**DOI:** 10.1016/j.redox.2025.103507

**Published:** 2025-01-20

**Authors:** Miltha Hidalgo, Bárbara Railef, Vania Rodríguez, Carolina Navarro, Vanessa Rubio, Jorge Meneses-Pacheco, Sandra Soto-Alarcón, Christine Kreindl, Carolina Añazco, Leandro Zuñiga, Omar Porras

**Affiliations:** a Laboratory for Research in Functional Nutrition, Instituto de Nutrición y Tecnología de los Alimentos, Universidad de Chile, Av. El Líbano 5524, Macul, Santiago, 7830490, Chile; b Nutrition and Dietetics, Faculty of Health Sciences, Universidad Autónoma de Chile, Santiago, 7500912, Chile; c Nutritional Biochemistry Laboratory, Faculty of Science for Health Care, Universidad San Sebastian, Valdivia, Chile; d Centro de Nanomedicina, Diagnóstico y Desarrollo de Fármacos (ND3), Laboratorio de Fisiología Molecular, Escuela de Medicina, Universidad de Talca, Casilla, Talca, 3460000, Chile

**Keywords:** CAPE, Caffeic acid, TRPV1, Endothelial cells, HyPer

## Abstract

Caffeic acid phenethyl ester (CAPE) is a hydrophobic phytochemical typically found in propolis that acts as an antioxidant, anti-inflammatory and cardiovascular protector, among several other properties. However, the molecular entity responsible for recognising CAPE is unknown, and whether that molecular interaction is involved in developing an antioxidant response in the target cells remains an unanswered question. Herein, we hypothesized that a subfamily of TRP ion channels works as the molecular entity that recognizes CAPE at the plasma membrane and allows a fast shift in the antioxidant capacity of intact endothelial cells (EC).

By monitoring cytoplasmic Ca^2+^ in a microvascular EC model, we compared the calcium responses evoked by three structurally related compounds: caffeic acid phenethyl ester, neochlorogenic acid and caffeic acid. Only CAPE induced rapid and transient calcium responses at nanomolar concentrations together with a gradual increase in cytoplasmic sodium levels, suggesting the activation of a non-selective cationic permeation at the plasma membrane. Electrophysiological as well as pharmacological, and RNA silencing assays confirmed the involvement of TRPV1 in the recognition of CAPE by ECs. Finally, we demonstrated that Ca^2+^ influx by TRPV1 was necessary for recording CAPE-induced cytoplasmic redox changes, a phenomenon captured in real-time in ECs expressing the HyPer biosensor.

Our data depict a molecular mechanism behind the antioxidant effect of CAPE in endothelial cells, connecting the activation of TRPV1 ion channels, cytoplasmic calcium increase, and a reduction of disulfide bonds on a redox biosensor. This phenomenon occurs within seconds to minutes and contributes to a better understanding of the mechanisms underlying the vasodilatory effect of CAPE and other compounds that interact with TRPV1 in the vascular bed.

## 1 Introduction

Caffeic acid phenethyl ester (CAPE) is a natural compound present in exudates from poplar [1] and the bark of coniferous trees, and it is harvested by honeybees as a component of propolis [2]. Since its identification in 1987, many biological activities and the therapeutic potential of CAPE have recently been reviewed [3,4].

Among the best-known effects of CAPE are its anti-inflammatory [[5], [6], [7], [8], [9], [10]] and antioxidant properties [[11], [12], [13]]. However, as observed in several animal models, CAPE also exhibits positive vascular effects on hypertension. For instance, Selamoglu's team and others have reported that both intraperitoneal injection of CAPE and propolis administration via gavage were effective in diminishing blood pressure in a rat model of hypertension induced by chronic inhibition of endothelial nitric oxide synthase with Nω-nitro-l-arginine methyl ester [[14], [15], [16]]. Similar findings were reported in hypertensive rats fed with chow containing 4 % NaCl and later treated with soluble propolis [17], as well as in dexamethasone-induced hypertensive rats treated with an apitherapy mix containing propolis, royal jelly and bee venom [18]. A recent meta-analysis covering seven studies showed that propolis consumption reduces systolic blood pressure in healthy subjects compared to the control group [19]. Despite all such evidence, most studies have explored the effect of CAPE within weeks of exposure, but only a few have investigated the immediate effect on the vascular bed. Among these few studies, a vasorelaxant effect of CAPE was observed in a matter of minutes in KCl or phenylephrine-treated pre-contracted rat aortic rings with an EC_50_ of 4.4–5 μM [20]. In the same temporal window, Burgazli et al. showed rapid calcium responses and stimulation of nitric oxide (NO) production in HUVEC cells exposed to CAPE [21]. It is worth mentioning that the physiological relevance of endothelial Ca^2+^ dynamics in vasodilatory responses has been elegantly demonstrated in mice expressing the Ca^2+^ sensor GCaMP2 predominantly in arterial ECs [22].

The evidence that supports the contribution of TRP channels in the function of the endothelium is growing. Of the 28 encoded proteins that comprise the TRP family, at least 10 have been found in ECs [23,24]. However, two members of the vanilloid subfamily of TRP channels - TRPV1 and TRPV4- have a role in vasodilation in small resistance arteries by promoting Ca^2+^ signaling that leads to an increment in NO production [[25], [26], [27], [28]], and in the case of TRPV4 also by promoting endothelial hyperpolarization via activation of IKCa and SKCa currents [29,30]. TRPV1 and TRPV4 can also recognise dietary compounds such as capsaicin [31], 8-shogaol [32], gingerols [33], apigenin [34], silybin [28] and eugenol [35], for instance. Therefore, as CAPE is also a dietary compound, it is reasonable to hypothesize that the aforementioned TRPV channels mediate CAPE's vascular effects.

This work shows that CAPE activates non-selective cationic currents sensitive to TRPV1-specific pharmacological blocking, leading to rapid Na^+^ and Ca^2+^ influxes in human dermal endothelial cells. In addition, we demonstrated that CAPE-induced TRPV1 activation not only promoted an elevation of cytoplasmic calcium but generated Ca^2+^ spikes, which disappeared by genetic silencing of TRPV1. Further experiments showed that TRPV1 activation and the concomitant calcium influx led to an increase in the reducing power of EC cytoplasmic proteins in a matter of seconds, according to real-time imaging of HyPer biosensor. This fluorescent redox biosensor changes its signal depending on the thiol/disulfide state of critical cysteine residues in its structure. Our data indicate that the antioxidant property of CAPE on endothelial cells is mediated by TRPV1 stimulation, offering a novel molecular mechanism that explains the interaction between botanical compounds and endothelial redox responses.

## 2 Materials and methods

**Fig. 1:**
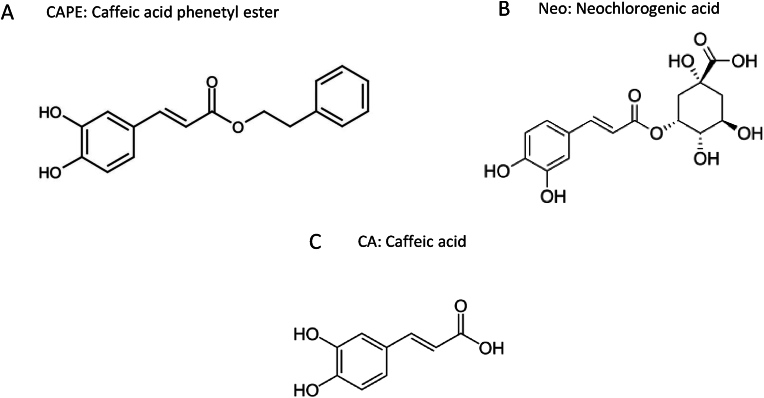
Chemical structures for Caffeic Acid Phenetyl Ester (CAPE, A), Neochlorogenic Acid (Neo, B) and Caffeic Acid (CA, C).

Reagents and salts for buffer preparation were obtained from Sigma-Aldrich (MO, USA). Media culture and supplements were purchased from ScienceCell Research Laboratories (C.A., USA) or American Type Culture Collection (ATCC, VA, USA). Ionophores, ion channel blockers and agonists were acquired from Tocris (MO, USA). Ethylene glycol bis (2-Aminoethyl ether)-N,N,N′,N′ tetra acetic acid (EGTA) and other reagents were acquired from Sigma-Aldrich (MO, USA). Fura-2 AM, SBFI-AM, Hoechst 33342 and pluronic acid were obtained from Thermo Fisher Scientific (M.A., USA). Caffeic acid phenethyl ester (CAPE) was purchased from Tocris (Bristol, UK), and caffeic acid (CA) and neochlorogenic acid (NEO), both were acquired from Extrasynthese (Genay, France); their chemical structures are displayed in Fig. 1. Stock solutions were prepared in DMSO at 10 mM. The 30 % hydrogen peroxide solution used in this work was from Merck (Darmstadt, Germany).

### 2.1 Cell culture

Endothelial cells (ECs) from human dermal microvasculature were acquired from the ATCC (CRL-4025™ TIME cells). Cultures were maintained following the provider's instructions. In general, cells were kept under a humidified atmosphere with 5 % CO_2_/air, and the culture medium was renewed every 2 or 3 days. When 70–80 % confluence was reached, cultures were expanded to other plates or seeded on glass coverslips (Marienfeld, Germany) to be further imaged. Cells were used in passages 6–12.

### 2.2 Ca^2+^ and Na ^+^ cell imaging

For imaging experiments, the coverslips were mounted in an open recording chamber, and media were replaced by KRH buffer (in mM: 140 NaCl, 4.7 KCl, 20 HEPES, 1.25 MgSO_4_, 1.25 CaCl_2_; pH 7.4), supplemented with 5 mM glucose (KRH-glc). Dyes were prepared in 0.01 % pluronic acid in DMSO solution; cells were loaded with 5 μM of the selected dye for 30 min at room temperature in KRH-glc. After that time, cells were rinsed three times with KRH-glc and left for another 20 min to allow complete de-esterification of the acetoxymethyl ester motif. Free-Ca^2+^ buffer was prepared as KRH-glc with nanopure water without CaCl_2_ but adding 5 mM EGTA. Typically, [Ca^2+^] in nanopure water ranges from 0.25 to 1.25 μM. If the Ca^2+^ concentration in the water for preparing the solutions were in the upper range, the expected free Ca^2+^ concentrations in the solution would be 17.6 pM (pH 7.4 at 25 °C), considered nominal calcium. Free calcium concentration was calculated using Max-Chelator (Chris Patton, Stanford University, CA, USA).

Imaging took place in an inverted Nikon Ti microscope equipped with a 40X oil objective (numerical aperture, N.A. 1.3). A xenon lamp was coupled to the monochromator device (Cairn Research Ltd, Faversham, U.K.), allowing dual excitation at 340 and 380 nm for Fura-2 and SBFI. A long-pass filter collected emission over 520 nm, and fluorescence was digitalized by a cooled CCD camera ORCA 03 (Hamamatsu, Japan), and analysis was performed using free micromanager software [36]. Data sampling was acquired every 20 s and expressed as the 340/380 ratio.

### 2.3 HyPer biosensor imaging

The HyPer biosensor was introduced into the cytoplasm of ECs by infection with adenovirus at 1: 100 dilutions. Our group has explained adeno-particle production in detail [37]. Briefly, pC1-HyPer-3 plasmid, a gift from Vsevolod Belousov (Addgene plasmid # 42131; http://n2t.net/addgene:42131; RRID: Addgene_42131) was sub-cloned into the commercial adenoviral vector pAdEasy-RFP using conventional molecular biology techniques, and homologous recombination was done using BJ5183 cell transformation. AdHek cells were used to expand and replicate viral particles. After 21 days of infection, cells were harvested and subjected to three freeze-thaw cycles, followed by centrifugation to remove cellular debris. The resulting supernatant (2 mL) was stored at −80 °C or used to infect target cells.

HyPer was homogeneously expressed in the cytosol two days after infection and ready for imaging. The biosensor was excited at 420 and 490 nm, whereas the emitted light was collected with a Long Pass filter over 520 nm. In our hands, the baseline in ECs showed stability over a lapse of 20 min, enough to detect redox shifts induced by phytochemicals.

### 2.4 Electrophysiological recording in TIME cells

Macroscopic currents obtained from TIME cells were studied using the whole-cell patch-clamp recording described elsewhere [38] using a PC-501A amplifier (Warner Instruments, Hamden, CT, USA). The voltage pulse generator and analysis programs were from Axon Instruments (Molecular Devices, LLC., CA, USA). Patch-clamp pipettes had resistances of 3–5 MΩ. The pipette solution in mM: 140 KCl, 1 CaCl_2_, 1 MgCl_2_, 10 glucose, 10 HEPES and pH was adjusted to 7.4 with KOH. The bath solution contained in mM: 140 NaCl, 5 KCl, 1 MgCl_2_, 2 CaCl_2_, 10 glucose, 10 HEPES; 7.4 was adjusted with NaOH. To isolate TRPV1-mediated currents from endogenous sodium currents, the extracellular solution was supplemented with tetrodotoxin (TTX) at 0.1 mM. Cells were held at 0 mV. Then, a ramp of 1000 ms of durations was applied from −100 to +100 mV, followed by a step to 0 mV. We adjusted the [CAPE] to 1 μM to avoid cellular collapse in patch-clamped cells and ensure complete recording during the ramp. The BCTC was used at 500 nM, which is 15–30 fold over the IC_50_ reported for TRPV1 [IC_50_ 17 nM [39]; IC_50_ 34 nM [40] and is below the IC_50_ value of 800 nM found for TRPM8 [41]. Perfusion of solutions into the extracellular bath of TIME cells was carried out before initiating electrophysiological recordings.

### 2.5 TRPV1 silencing

Specific siRNA for human TRPV1 (Cat. #4392420) and the negative control siRNA (Cat. #4390843) were purchased from Thermo Fisher Scientific (MA, USA). Before cell transfection, the siRNAs were labelled using the Silencer™ siRNA Labeling Kit with Cy™3 dye (AM4621, Invitrogen, USA) following the manufacturer's instructions. Briefly, 20 μl of siRNA (20 μM) were incubated at 37 °C for 1 h in the presence of 5 μl of 10X labelling buffer, 20 μl of nuclease-free water, and 5 μl of labelling dye. Subsequently, 5 μl of NaCl (5 M) and 125 μl of absolute ethanol (100 %) were added. The mixture was incubated at −20 °C for 30 min, then centrifuged at maximum speed (4 °C, 20 min), and the supernatant was discarded. Finally, the labelled siRNA pellet was air-dried and resuspended in 20 μl of nuclease-free water, resulting in siRNA ready for transfection.

The siRNA transfection was performed in TIME cells using the siPORT™ NeoFX™ transfection agent (Cat. #AM4511, Invitrogen, USA). Concisely, 5 μl of siPORT NeoFX transfection agent were diluted in Opti-MEM medium (Gibco-Invitrogen, USA) to a total volume of 100 μl and incubated at room temperature for 10 min. In parallel, siRNAs were diluted in 100 μl of Opti-MEM medium to achieve a final concentration of 30 nM in the cell culture medium. The diluted siRNAs and transfection agent solutions were combined, incubated for 10 min, and dispensed into culture plates at a final volume of 1.5 ml per well. The cells were incubated for 48 h before further assays.

### 2.6 Statistical analysis

Throughout the manuscript, data are expressed as mean ± standard error. Particularly for single-cell recordings obtained with imaging experiments, Paired Student's t-tests were executed to compare 3-min data bins before and after treatments. ANOVA tests were used for more than two group comparisons, and repeated measurements were evaluated by ANOVA with Bonferroni *post hoc* analysis for parametric data or Dunn's method for non-parametric data when corresponding. A *p*-value less than 0.05 was considered statistically significant.

## 3 Results

### 3.1 CAPE triggers fast calcium responses in TIME cells

**Fig. 2:**
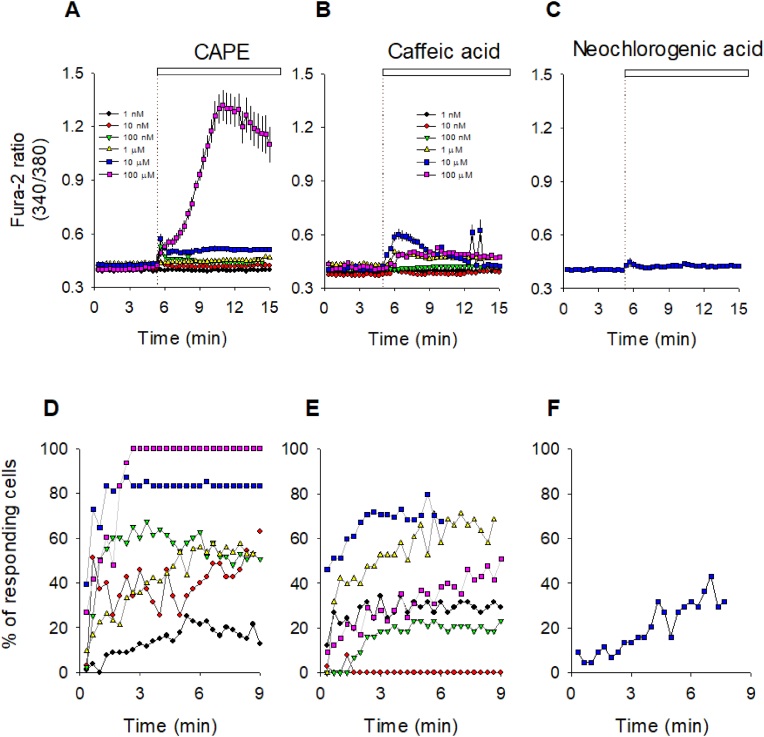
Time-courses of calcium responses evoked by CAPE, Caffeic and Neochlorogenic Acids and the percentage of responding endothelial cells. TIME cells were loaded with Fura-2 and exposed to a range of concentrations from 1 nM to 100 μM. After 5 min of stable baseline; phytochemicals were added as indicated by dotted lines and white boxes on the upper plots **A**, **B** and **C**. Since the effect of neochlorogenic acid on TIME cells was discrete, we only show the result obtained at 10 μM. Data was collected every 20 s and is expressed as average ± SE of 35–138 single-cell recordings from at least three independent experiments. The percentage of responding cells over time is in the lower plots **D**, **E** and **F**. Cells were considered responders if Fura-2 ratio values were higher than the average baseline plus three times the SD value.

TIME cells were loaded with the fluorescent calcium indicator Fura-2 and exposed to three structurally related phytochemicals that share the caffeic acid moiety (3,4-dihydroxycinnamic acid). This moiety is either conjugated with a tetrahydroxy-cyclohexane ring that confers water solubility to neochlorogenic acid or esterified with the phenethyl group that made CAPE hydrophobic (see Fig. 1). These physicochemical differences are determinants for effective interaction with cellular components such as protein carriers, ion channels or the plasma membrane. Among the three selected compounds, we found chlorogenic acid ineffective in inducing calcium responses compared to caffeic acid and CAPE (Fig. 2). We observed transient increases in cytoplasmic calcium at nanomolar concentrations with CAPE, as can be appreciated in Supplemental Fig. 1, where all the single-cell recordings are presented. For example, roughly 50 % of the imaged cells showed a positive calcium signal with 10 nM CAPE. In contrast, at the same concentration, no more than 10 % of the cell population increased their cytoplasmic calcium in the presence of caffeic acid. As expected, the proportion of responding cells increased according to the concentration of CAPE and caffeic acid augmented (Fig. 2D and E). Although all three compounds evaluated here share the caffeic acid moiety, it is noteworthy that the hydrophobicity of this molecule is crucial for effective interaction with cellular components, likely at the plasma membrane, which ultimately determines its bioactivity since the most hydrophilic compound, chlorogenic acid, only evoked a very discrete response at 10 μM (Fig. 2C).

**Fig. 3:**
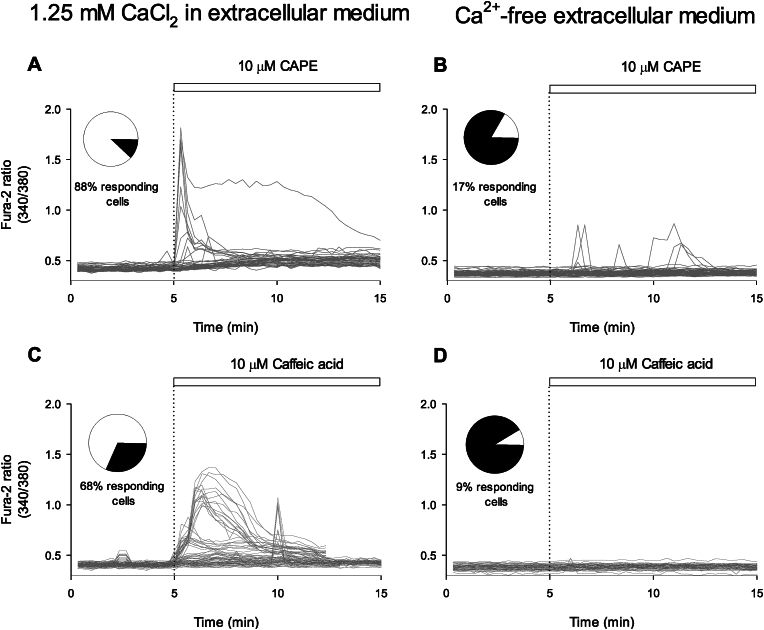
Extracellular Ca^2+^ removal decreases the magnitude of calcium responses and the number of responding ECs induced by CAPE and Caffeic Acid. After loading TIME cells with Fura-2, cells were exposed to 10 μM CAPE (**A**) or caffeic acid (**C**) at the time indicated by dotted lines and the white bars on the plots. **B** and **D**, the experiments were carried out without extracellular Ca^2+^ with a buffer supplemented with 5 mM EGTA. Data acquisition was made every 20 s, and each single-cell recording was shown as a grey trace (48–82 imaged cells from at least four independent experiments). The pie chart inserted in each plot illustrates the proportion of responding cells (white) and non-responding cells (black).

The extracellular origin of the calcium responses induced by CAPE and caffeic acid was established by running calcium imaging experiments on TIME cells kept in an extracellular buffer without Ca^2+^. In Fig. 3, rapid elevations in cytosolic calcium levels can be observed upon adding 10 μM CAPE and caffeic acid (Fig. 3). The removal of this divalent cation from the medium avoided most of the responses to CAPE or caffeic acid seen in TIME cells; only a minor cell population presented a calcium increase, which is close to the 11 % of spontaneous activity that we observed in control experiments with 0.01 % DMSO (Supplemental Fig. 2).

**Fig. 4:**
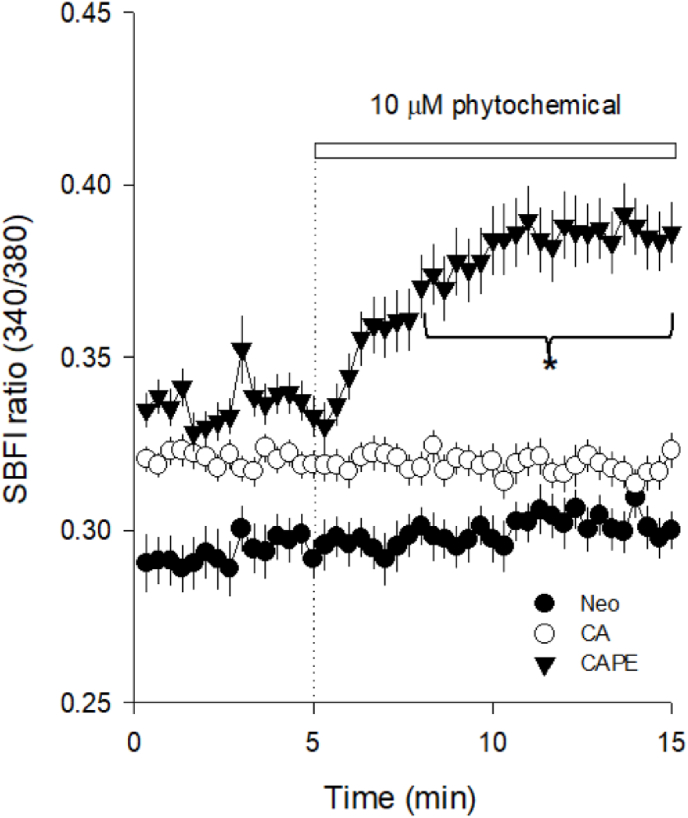
Only CAPE induces an increase in cytoplasmic Na^+^ in endothelial cells. TIME cells were loaded with SBFI, a ratiometric fluorescent Na^+^ indicator. At the time indicated by the dotted line and white bar on the plot, 10 μM of CAPE (black triangles), caffeic acid (CA, empty circles) and neochlorogenic acid (Neo, full circles) were added. Cell imaging sampling was acquired every 20 s, and data were expressed as the average ± SE from 20 to 58 cells imaged in at least three independent experiments. The asterisk represents statistical differences compared to the baseline obtained by RM-ANOVA Dunn's post hoc.

To characterize the nature of permeation via activation by these phytochemicals, we hypothesized that the activation of non-selective cationic ion channels at the plasma membrane could mediate the Ca^2+^ entry in these microvascular endothelial cells. If this were the case, we would expect that TIME cells become Na ^+^ loaded upon stimulation with the phytochemicals that promoted an elevation of cytoplasmic Ca^2+^. With this purpose in mind, we loaded the cells with SBFI dye, a ratiometric fluorescent Na^+^ indicator, to track intracellular level fluctuations of this monovalent ion. Only CAPE (10 μM) induced a gradual increase in SBFI signal that significantly differed from basal values after the third minute of exposure (Fig. 4).

**Fig. 5:**
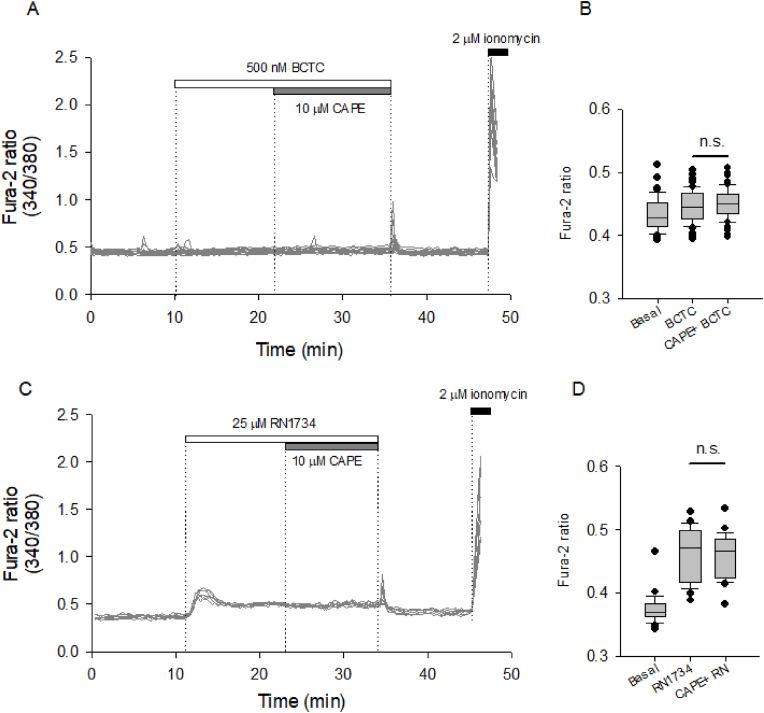
Pharmacological blockage of TRPV1 and TRPV4 interfered with the calcium responses evoked by CAPE in TIME cells. **A**, a time course of calcium imaging performed on TIME cells loaded with Fura-2. After 10 min of stable baseline, 500 nM BCTC was added, as indicated by a dotted line and the white box on the plot. Subsequently, 10 μM CAPE was added to cells, indicated by another dotted line and a grey box on the plot. Finally, 2 μM ionomycin was used at the end of the recordings to corroborate that the dye was sensitive to a Ca^2+^ increase. Here, 12 single-cell recordings (grey lines) from a representative experiment are presented. In **B**, a summary of 64 single-cell recordings obtained from four independent experiments is represented by box plots where 3-min bins of basal, in the presence of BCTC and BCTC + CAPE, were averaged to facilitate the comparison. In **C**, a representative experiment with seven single-cell recordings, from a representative experiment, shows the effect of 25 μM RN1734 on resting levels of cytosolic. After 10 min, 10 μM CAPE was added. The experimental manipulations are indicated in the same manner as in A. In **D**, like B, a summary of 24 single-cell recordings is represented by box plots. RM-ANOVA, followed by a Tukey Test, was performed to compare multiple groups.

**Fig. 6:**
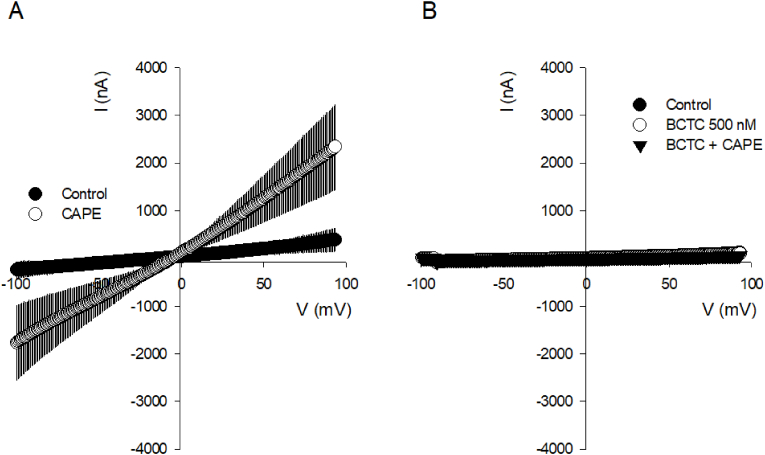
CAPE induces the activation of BCTC-sensitive non-selective cationic currents in TIME cells. Current-voltage relationship obtained from TIME cells studied in whole-cell mode. **A.** The average ± SE of current families obtained from 5 independent experiments are shown, with basal current indicated by a solid circle (control) and the current following CAPE perfusion indicated by an open circle. **B**, The average I/V curve under control conditions (basal current, indicated by a solid circle) and in the presence of BCTC (open circle) and BCTC plus CAPE (closed triangle). Data correspond to the average ± SE from 3 independent experiments.

At this point, 10.13039/501100024956CAPE triggers the influx of Na^+^ and Ca^2+^ from the extracellular space, which supports the plausible participation of TRPV1 and TRPV4 channels, both non-selective cationic channels involved in endothelial-mediated vasodilation and well-known for their capacity to interact with phytochemicals. To test this possibility, TIME cells were pre-treated with BCTC and RN1734, both pharmacological blockers for TRPV1 and TRPV4, respectively, and then exposed to 10 μM CAPE (Figure 5A-5C). Our recordings showed that 0.5 μM BCTC effectively blocked the CAPE-induced calcium increases. Curiously, the addition of 25 μM RN1734 generated an immediate increase in the resting calcium levels, which exhibited a moderate recovery but was insufficient to bring cytosolic calcium back to basal levels (Fig. 5C); unfortunately, this perturbation in the intracellular calcium state could mask any further actions of CAPE (Fig. 5D). The further involvement of TRPV1 in the Ca^2+^ and Na ^+^ influx induced by CAPE was assessed through whole-cell patch-clamp experiments in TIME cells. Applying 1 μM CAPE at the bath solution increased the currents observed during a ramp between −100 and 100 mV (Fig. 6A), indicating that CAPE triggers a voltage-independent non-selective cationic channel opening that, in addition, was sensitive to the pharmacological blockage of TRPV1 with BCTC (Fig. 6B).

**Fig. 7:**
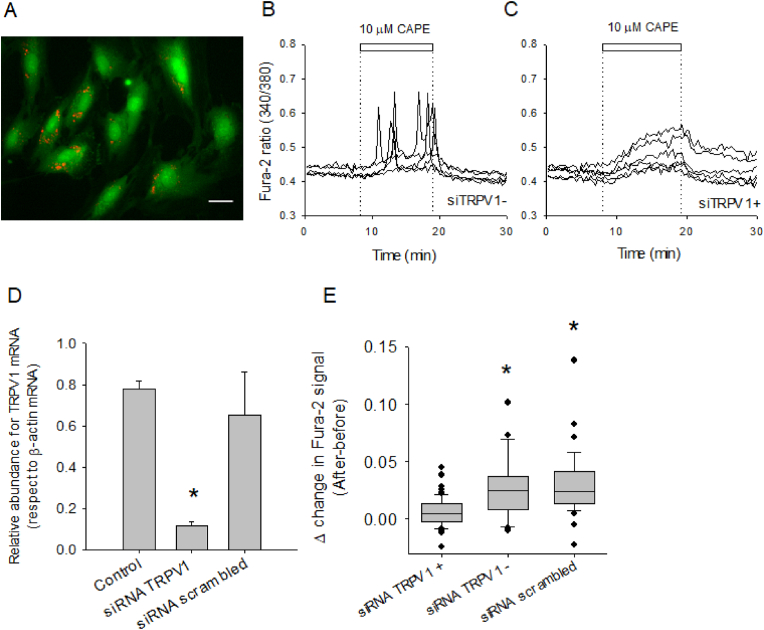
Silencing of TRPV1 channels modifies the kinetics and intensity of CAPE-induced calcium responses in TIME cells. **A**, microphotography of Fura-2 loaded-TIME cells in which some cells show intracellular red dots indicating the positive transfection of Cy3-labelled oligo RNAs directed against TRPV1 mRNA. The white bar in the image indicates 20 μm. **B**, single-cell recordings of intracellular calcium levels of five cells without red dots (siTRPV1-). The dotted line and white bar indicate when 10 μM CAPE was added. **C**, as in B, six single-cell recordings of cells that present red dots in their cell bodies (siTRPV1+). In **D**, the relative abundance of mRNA for TRPV1 in untreated TIME cells (control, five samples), transfected with oligo RNAs directed against TRPV1 (siRNA TRPV1, ten samples) and transfected with an oligo RNA with a scrambled nucleotidic sequence (siRNA scrambled, three samples). The abundance of β-actin mRNA was used as the housekeeping gene. Data are expressed as average ± SE, and the asterisk corresponds to the one-way ANOVA (Dunn's Method). **E**, a comparison of the calcium responses evoked by 10 μM CAPE, expressed as the difference between baseline and after 3 min in the presence of CAPE, in three groups of cells, those which were successfully transfected (siTRPV1+), those without signs of transfection (siTRPV1-) and cells transfected with the scrambled siRNA.

Based on the clean blockage observed with BCTC, we decided to knock down the expression of TRPV1 by silencing the gene post-transcriptionally with specific siRNA for TRPV1 messenger RNAs and test the capacity of CAPE to induce calcium increases in TRPV1-siRNA cells. By labelling the RNA oligos with a Cy3 fluorescence, we observed roughly 80 % of the cell population with intracellular red dots, indicating a high efficiency of transfection with siRNA (101 transfected cells out of 127 cells observed in five independent experiments) (Fig. 7A). With this approach, we could simultaneously compare calcium responses evoked by 10 μM CAPE on a mixed population of TIME cells, one without red fluorescence, siTRPV1- (Fig. 7B) and another with intracellular red spots due to cytoplasmic Cy3-labelled siTRPV1+ (Fig. 7C). By comparing both plots, it is evident that cells that incorporated the siRNA against TRPV1 did not show calcium peaks as observed in cells without red dots. However, we noted a gradual increase in the cytoplasmic calcium during the extracellular presence of CAPE; this cellular response is reversible since after the removal of the phytochemical, the calcium signal started to diminish to basal levels. We confirmed the efficiency of siRNA against TRPV1 mRNA by evaluating the relative abundance of the mRNA TRPV1 after 48 h of transfection (Fig. 7D). Finally, the analysis of the calcium response intensities during the first 3 min of exposure to CAPE indicated that cells that incorporated the siRNA against TRPV1 had feeble responses compared to those cells without red dots or subjected to transfection with a scrambled oligo RNA sequence (Fig. 7E).

### 3.2 The antioxidant properties of CAPE depend on the activation of TRPV1 in the endothelial TIME cells

**Fig. 8:**
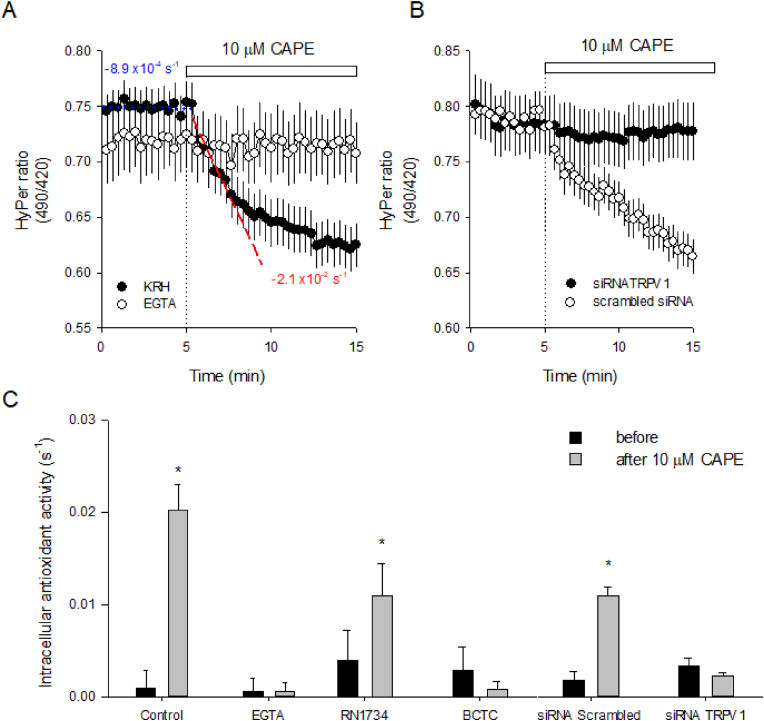
The antioxidant effect of CAPE depends on TRPV1 and is mediated by the Ca^2+^ influx in endothelial cells. **A**, HyPer-expressing TIME cells were subjected to 10 μM CAPE in the presence of extracellular Ca^2+^ (full circles) or the absence of this cation by using a buffer without Ca^2+^ and supplemented with 5 mM EGTA (empty circles). The dotted line and white bar on the plot indicate the moment when CAPE was added. Data correspond to the average ± SE of 32–53 single-cell recordings from 4 to 6 independent experiments. Linear regression was performed on baseline data (5 min), depicted in a dotted blue line, and on the same period after CAPE exposure, illustrated by a discontinued red line. The resulting basal and CAPE-stimulated slopes are on the plot to describe how the intracellular antioxidant activity was estimated. In **B**, HyPer-expressing TIME cells were transfected with oligo RNAs directed against TRPV1 mRNA (siRNA TRPV1) or scrambled oligo RNA (scrambled siRNA) were exposed to 10 μM CAPE at the time indicated by the dotted line and white bar. Data correspond to the average ± SE of 16–26 single-cell recordings from 3 independent experiments. In **C**, a summary of the slopes of linear regressions obtained from baselines (before) and after the addition of CAPE in the control situation, without extracellular Ca^2+^, in the presence of TRPV antagonists, RN1734 and BCTC, and on cells transfected with scrambled siRNA and siRNA TRPV1. Data correspond to the average ± SE of the slopes obtained from the single-cell recordings declared in A and B. For the TRPV antagonists, 12–28 recordings were averaged. In all the cases, more than three independent experiments were performed. The asterisk indicates the significant difference between before and after groups obtained by paired t-student test.

Our next step was to monitor the antioxidant capacity of TIME cells by introducing the fluorescent redox biosensor HyPer into their cytoplasm. The fluctuations in the signal of this biosensor report the reversible and dynamic nature of thiol/disulfide interchange that occurs in the cytoplasm of living cells. With this molecular tool, we detected a fast drop in the signal of this redox biosensor induced by 10 μM CAPE; such spectral change showed a strong dependence on the presence of Ca^2+^ in the extracellular medium since CAPE did not modify the HyPer signal when the experiments were done with an extracellular buffer without Ca^2+^ and supplemented with EGTA, a chelator of this divalent cation (Fig. 8A). To measure the magnitude of this redox phenomenon, we considered 5 min of data before and after CAPE exposure to compare the CAPE-induced increase in the antioxidant capacity of the cytoplasm. With this analysis, the slope estimated for the period before CAPE addition (in blue, Fig. 8A) experienced a remarkable increase, more than one order of magnitude, in the presence of CAPE (in red, Fig. 8A). Next, we tested if the CAPE-induced redox effect was observable in TIME cells subjected to TRPV1 expression interference. As shown in Fig. 8B, CAPE did not affect the redox state of HyPer biosensor-expressed TIME cells treated with the siRNA against TRPV1. Here, it is important to mention that we did not label the siRNAs; therefore, we were blind to positively transfected cells during HyPer imaging, and based on our experience, we estimated that 20 % of non-transfected cells were not enough to mask the effect of TRPV1 interference. As expected, TIME cells transfected with a scrambled sequence of RNA were redox-sensitive to the addition of CAPE. Consistent with this observation, the addition of BCTC avoided the redox effect of CAPE, whereas RN1734 did not interfere with the effect of CAPE on the biosensor (Fig. 8C and Supplemental Fig. 3). On the other hand, capsaicin and GSK1016790A, specific agonists for TRPV1 and TRPV4, respectively, mimicked the redox effect observed with CAPE, although capsaicin presented a higher antioxidant effect than GSK1016790A (Supplemental Figs. 3B–C). Our data suggests that CAPE triggers an intracellular redox effect mainly mediated by the activation of TRPV1 channels.

## 4 Discussion

The antioxidant properties of dietary phytochemicals are commonly associated with potential health benefits. This notion might create high expectations, even when the molecular interactions between dietary compounds and mammalian cells have not been fully described. Here, we demonstrated that caffeic acid phenethyl ester, a compound found in honeybee products with multiple bioactivities, exerts its antioxidant effects by activating TRPV1 channels in microvascular human endothelial cells.

We chose three structurally related molecules that carry the hydroxycinnamic acid moiety in their structures with different hydrophobicity indexes (www.molinspiration.com): Neochlorogenic acid, which brings a polyol cyclohexane ring, is the most polar with an index of −0.45, whereas caffeic acid phenethyl ester is the most hydrophobic of them with an estimated index of 3.36. The catechol moiety and the hydrophobicity that characterize CAPE might be fundamental for interaction with non-selective cationic channels like TRPVs in endothelial cells. The protein structure of TRPV1 achieved by Cryo-EM indicates that capsaicin finds a hydrophobic pocket formed by S3–S4 helixes modulating the channel gating [42]. The affinity of the TRPV1 channel for hydrophobic compounds is supported by the activation induced by lysophosphatidic acid in membrane patches from TRPV1-expressing HEK cells and DRG neurons and the inhibition exerted by oleic acid on the activation of this channel by voltage and capsaicin [43,44]. In the work of Paracatu et al., these three compounds were evaluated in several cell-free assays, such as H_2_O_2_ reactivity, DDPH scavenging and triene degradation, with no differences found between them. However, they found that CAPE was the only compound inhibiting ROS production in activated human lymphocytes and neutrophils [45]. Similar to our observations, other groups have also reported that CAPE is more effective than caffeic acid in inhibiting ROS production in human erythrocyte membranes [46] and protecting against H_2_O_2_-induced DNA damage in HeLa cells [47]. Our results place CAPE as another hydrophobic compound able to interact allosterically with TRPV1, activating the channel independently of cysteine residue modification, as was demonstrated for garlic and onion extracts by Salazar et al. [48].

### 4.1 Intracellular redox shift mediated by TRPV1 stimulation

Real-time imaging of HyPer3 allows the observation of reversible redox fluctuations in the cytoplasm of intact endothelial cells. The HyPer fluorescent signals fluctuate according to the balance between the thiol/disulfide ratio of critical cysteine residues in the biosensor structure. Therefore, the kinetics of HyPer can be employed to follow the production of intracellular H_2_O_2_ induced by stimulation of growth factor receptors [49] and monitor the rate of disulfide bond reduction by the cellular antioxidant systems [37,50,51]. Upon CAPE exposure, we observed an immediate drop in the biosensor signal, an effect abolished when the experiment was carried out without extracellular Ca^2+^ and when the TRPV1 channels were pharmacologically blocked with BCTC or knocked down by silencing their mRNAs, indicating that calcium influx through this ion channel was a necessary step to activate the intracellular machinery responsible for reducing disulfide bonds.

Among the elements constituting the antioxidant machinery in mammalian cells, thioredoxin-1 (Trx-1) is key in reducing HyPer biosensor when expressed at the cytoplasm, as recently demonstrated by Zhuravlev et al. [51]. Nevertheless, Trx-1 did not appear among the 2884 calcium-regulated proteins found by thermal stability changes upon the addition of Ca^2+^ [52]. Other regulatory mechanisms, such as S-nitrosylation on specific cysteines on Txr-1, have been demonstrated to be fundamental for the regulation of its reducing activity and anti-apoptotic function [53,54]. Dynamic changes in the S-nitrosylation state of Txr-1 have been reported to occur in the range of seconds to minutes in *C. elegans* as part of a mechanism to avoid nitric oxide donor microorganisms, a system that works coupled to calcium dynamics on chemosensory neurons of this organism [55]. Another regulator of Trx-1 activity is the thioredoxin-interacting protein TXNIP, an α-arrestin protein that regulates glucose and lipid metabolism [56,57]. TXNIP has been implicated in the fast stimulation of glucose uptake induced by growth factors on three different human cell lines, a phenomenon mediated by phosphorylation by AKT in 15 min after exposure to growth factors [58]. From the mentioned regulatory mechanisms operating on Trx-1 activity, S-nitrosylation has some attractiveness based on calcium dynamic coupling. Beyond the potential mechanisms that could link TRPV1 activation with the rapid increase in the antioxidant capacity of the cytoplasmic environment of microvascular ECs, it remains necessary to elucidate how the cells discriminate different patterns in calcium signaling (spikes, waves, sparklets, etc.) to execute a specific cellular response.

### 4.2 TRPV1 channels in endothelial cells and vasodilatory action

We tested the contribution of TRPV1 and TRPV4 in the endothelial redox response induced by CAPE, both recognized by promoting Ca^2+^-dependent vasorelaxation [30,59]. Observing calcium dynamics with sophisticated techniques has revealed a complex interplay between calcium influx through ion channels [60] and calcium release from internal storage [22,61]. In addition to this cellular scenario, the molecular mechanisms orchestrating Ca^2+^ dynamics and vasodilation depend on the specifics of the vascular beds. In recent work, Zhang et al. demonstrated that capsaicin-induced vasodilation was predominantly governed by TRPV1 in first-order mesenteric arteries (lumen diameter of >300 μm). In contrast, the same compound exerts its vasodilatory effect by TRPV4 in second-order branches (lumen diameter of <300 μm) [62]. Our study observed that TRPV1 was key for the CAPE-induced calcium responses to develop as a mixed pattern (i.e., sustained calcium increase with spikes). A recent study by Zuccolo et al. demonstrated that Ca^2+^ oscillations were coupled to NO production in microvascular endothelial cell lines from mouse and human brains [63]. Our data on TRPV1 silencing further unveils the importance of Ca^2+^ dynamics, characterized by spikes, in the disulfide reduction at the cytoplasm of EC.

The functional relevance of calcium dynamics for vascular function, considering the heterogeneity of peripheral territory, is still under active research. However, calcium imaging observations in small mesenteric arteries of TRPV4 KO mice (<100 μm) allowed the authors to propose that the level of activation of TRPV4 could determine the difference between physiological vascular adjustment and excessive blood pressure reduction with vascular permeability observed in septic shock, for instance Ref. [60].

Only a few reports have explored rapid responses evoked by CAPE in endothelial cells; among them, the work of Burgazli et al. was one of the first showing that CAPE induced fast increases in cytoplasmic Ca^2+^ and hyperpolarization in HUVEC cells with the concomitant augment in NO levels [21]. Accordingly, the intravenous injection of CAPE (5 mg/kg) induced a rapid decrease in the arterial pressure of animals, an effect that was independent of vagal activity [64] and not observable in denuded blood vessels, suggesting that the compound interacts with endothelial cells directly [20,65]. Additional evidence shows that CAPE promotes vasorelaxation in arterioles from pigs and rats and that a two-week treatment with CAPE (50 μmol/kg) was enough for recovery from elevated blood pressure in hypertense rats induced by a high fructose diet [20,65,66]. This effect was achieved by improving the expression of the endothelial isoform of nitric oxide synthase (eNOS) in aortic tissue at similar levels to the control group [66].

The role of TRPV1 channels in vasodilation of blood vessels has been established in mice and human arterioles [27,59,67]. From those studies, diverse stimuli that activate TRPV1 channels like hyperosmosis, dietary compounds (niacin, capsaicin) and hydrogen peroxide [68] lead to Ca^2+^ influx, which can ultimately stimulate nitric oxide production by promoting the phosphorylation of eNOS [69,70]. Curiously, in the thermally induced increase of skin blood flow, activation of TRPV1 channels plays a predominant role in rapid vasodilatory response that presents a peak within the first 3–5 min after thermic stimulus. This physiological response was significantly reduced by pharmacological blocking of eNOS yet not abolished [71,72]. Moreover, the blocking of both TRPV1 and eNOS was shown to be additive [71], indicating that heat-induced vasodilation is not exclusively mediated by stimulation of nitric oxide production. Perhaps the increase in the antioxidant capacity of the endothelial cytoplasm commanded by TRPV1 activation improves the bioavailability of this gaseous vasodilator, acting as a complementary mechanism during TRPV1-mediated vasodilation [73].

### 4.3 The anti-inflammatory role of TRPV1

Over 25 years ago, the capacity of CAPE to block the activation of NF-κB in U937 cells induced by the recombinant tumour necrosis factor (TNF) was reported by Natarajan et al. [74]. After this, many other studies confirmed the finding, expanding the scope to *in vivo* models for intestinal inflammation [9,[75], [76], [77]] and cigarette smoke-induced airway inflammation [78,79]. CAPE has been protective in experimental scenarios for inflammatory bowel diseases [77,[80], [81], [82]]. However, the first clues involving TRPV1 as part of the underlying mechanisms came from studies with capsaicin, which, at the micromolar range, showed anti-inflammatory action in murine macrophages and liver exposed to LPS [83,84] and on human keratinocytes from vitiligo lesions [85] and gastric epithelial cells exposed to *Helicobacter pylori* [86]. Whether the anti-inflammatory action of CAPE is mediated by TRPV1 activation has not been established yet. The cellular redox shift induced by CAPE reported in this work could stabilize inhibitory regulators of NF-κB, such as IKBKG (NEMO), a protein recently identified as redox-sensitive by thermal profiling proteomics in an unpublished preprint by Belousov's group [52].

The physicochemical properties of CAPE, such as its high hydrophobicity and the hydrolysable ester bond, may contribute to a limited bioavailability, a drawback considering the intake as the via of administration [87]. However, the broad spectrum of bioactivities associated with this compound, along with its differential stability observed in the plasma of human samples compared to rat plasma [88,89], constitute attractive elements for topic application and development of nanotechnology arrays. Wounds at the buccal cavity and skin are excellent examples of the topical application of this compound in gel format [90,91]. The nanoencapsulation of CAPE has allowed delivering this compound to the colon to ameliorate inflammation during active colitis [9]. For instance, increased solubility and drug release have been achieved by nanoencapsulation to treat lymphoma in animal models [91].

Our data shed light on the molecular mechanisms of CAPE in a cellular model for microvasculature, which reaches all of the body's peripheral regions and participates in appropriate wound healing and angiogenesis in tumour growth. We place TRPV1 as a molecular mediator in the calcium entry and redox effect of CAPE, which helps explain its vasodilatory and anti-inflammatory actions.

## CRediT authorship contribution statement

**Miltha Hidalgo:** Conceptualization, Data curation, Formal analysis, Investigation, Methodology, Supervision, Writing – original draft, Writing – review & editing. **Bárbara Railef:** Data curation, Formal analysis, Investigation, Methodology, Validation, Writing – original draft. **Vania Rodríguez:** Conceptualization, Investigation, Methodology, Writing – review & editing. **Carolina Navarro:** Conceptualization, Investigation, Methodology. **Vanessa Rubio:** Conceptualization, Investigation, Methodology. **Jorge Meneses-Pacheco:** Conceptualization, Formal analysis, Writing – original draft, Writing – review & editing. **Sandra Soto-Alarcón:** Conceptualization, Methodology, Writing – review & editing. **Christine Kreindl:** Conceptualization, Formal analysis, Investigation, Methodology. **Carolina Añazco:** Funding acquisition, Supervision, Writing – review & editing. **Leandro Zuñiga:** Conceptualization, Data curation, Formal analysis, Investigation, Validation, Writing – original draft, Writing – review & editing. **Omar Porras:** Conceptualization, Data curation, Formal analysis, Funding acquisition, Investigation, Methodology, Project administration, Resources, Supervision, Validation, Visualization, Writing – original draft, Writing – review & editing.

## Declaration of competing interest

The authors declare that they have no known competing financial interests or personal relationships that could have appeared to influence the work reported in this paper.

## References

[1] Greenaway W.T., Scaysbrook T., Whatley F.R. (1987). The analysis of bud exudate of Populus x euramericana, and of propolis, by gas chromatography–mass spectrometry. Proc. R. Soc. Lond. Ser. B Biol. Sci..

[2] Bankova V., Dyulgerov A., Popov S., Marekov N. (1987). A GC/MS study of the propolis phenolic constituents. Z. Naturforsch. C Biosci..

[3] Calabrese E.J., Pressman P., Hayes A.W., Baldwin L., Agathokleous E., Dhawan G. (2024). Caffeic acid: numerous chemoprotective effects are mediated via hormesis. J. Diet. Suppl..

[4] Bava R., Castagna F., Lupia C., Poerio G., Liguori G., Lombardi R. (2024). Hive products: composition, pharmacological properties, and therapeutic applications. Pharmaceuticals.

[5] Reddy A.M., Seo J.H., Ryu S.Y., Kim Y.S., Kim Y.S., Min K.R. (2004). Cinnamaldehyde and 2-methoxycinnamaldehyde as NF-kappaB inhibitors from cinnamomum cassia. Planta Med..

[6] Sticozzi C., Belmonte G., Meini A., Carbotti P., Grasso G., Palmi M. (2013). IL-1beta induces GFAP expression in vitro and in vivo and protects neurons from traumatic injury-associated apoptosis in rat brain striatum via NFkappaB/Ca(2)(+)-calmodulin/ERK mitogen-activated protein kinase signaling pathway. Neuroscience.

[7] Hong J.Y., Lee K.E., Kim K.W., Sohn M.H., Kim K.E. (2010). Chitinase induce the release of IL-8 in human airway epithelial cells, via Ca2+-dependent PKC and ERK pathways. Scand. J. Immunol..

[8] Papademetrio D.L., Lompardia S.L., Simunovich T., Costantino S., Mihalez C.Y., Cavaliere V. (2016). Inhibition of survival pathways MAPK and NF-kB triggers apoptosis in pancreatic ductal adenocarcinoma cells via suppression of autophagy. Targeted Oncol..

[9] Tambuwala M.M., Khan M.N., Thompson P., McCarron P.A. (2019). Albumin nano-encapsulation of caffeic acid phenethyl ester and piceatannol potentiated its ability to modulate HIF and NF-kB pathways and improves therapeutic outcome in experimental colitis. Drug Deliv Transl Res.

[10] Stahli A., Maheen C.U., Strauss F.J., Eick S., Sculean A., Gruber R. (2019). Caffeic acid phenethyl ester protects against oxidative stress and dampens inflammation via heme oxygenase 1. Int. J. Oral Sci..

[11] Marin E.H., Paek H., Li M., Ban Y., Karaga M.K., Shashidharamurthy R. (2019). Caffeic acid phenethyl ester exerts apoptotic and oxidative stress on human multiple myeloma cells. Invest. N. Drugs.

[12] Choi K., Han Y.H., Choi C. (2007). N-acetyl cysteine and caffeic acid phenethyl ester sensitize astrocytoma cells to Fas-mediated cell death in a redox-dependent manner. Cancer Lett..

[13] Song J.J., Lim H.W., Kim K., Kim K.M., Cho S., Chae S.W. (2012). Effect of caffeic acid phenethyl ester (CAPE) on H(2)O(2) induced oxidative and inflammatory responses in human middle ear epithelial cells. Int. J. Pediatr. Otorhinolaryngol..

[14] İlhan S., Yılmaz N., Nacar E., Motor S., Oktar S., Şahna E. (2014). The effect of caffeic acid phenethyl ester on isoproterenol-induced myocardial injury in hypertensive rats. Anadolu Kardiyol. Derg..

[15] Ekhteiari Salmas R., Durdagi S., Gulhan M.F., Duruyurek M., Abdullah H.I., Selamoglu Z. (2018). The effects of pollen, propolis, and caffeic acid phenethyl ester on tyrosine hydroxylase activity and total RNA levels in hypertensive rats caused by nitric oxide synthase inhibition: experimental, docking and molecular dynamic studies. J. Biomol. Struct. Dyn..

[16] Ozdemir B., Gulhan M.F., Sahna E., Selamoglu Z. (2021). The investigation of antioxidant and anti-inflammatory potentials of apitherapeutic agents on heart tissues in nitric oxide synthase inhibited rats via Nω-nitro-L-arginine methyl ester. Clin. Exp. Hypertens..

[17] Zhou H., Wang H., Shi N., Wu F. (2020). Potential protective effects of the water-soluble Chinese propolis on hypertension induced by high-salt intake. Clin Transl Sci.

[18] Abd El-Hakam F.E., Abo Laban G., Badr El-Din S., Abd El-Hamid H., Farouk M.H. (2022). Apitherapy combination improvement of blood pressure, cardiovascular protection, and antioxidant and anti-inflammatory responses in dexamethasone model hypertensive rats. Sci. Rep..

[19] Bahari H., Shahraki Jazinaki M., Goudarzi K., Namkhah Z., Taheri S., Golafrouz H. (2024). Effects of propolis consumption on blood pressure, lipid profile and glycemic parameters in adults: a GRADE-assessed systematic review and dose-response meta-analysis. Br. J. Nutr..

[20] Cicala C., Morello S., Iorio C., Capasso R., Borrelli F., Mascolo N. (2003). Vascular effects of caffeic acid phenethyl ester (CAPE) on isolated rat thoracic aorta. Life Sci..

[21] Kamil Burgazli M., Aydogdu N., Rafiq A., Mericliler M., Chasan R., Erdogan A. (2013). Effects of caffeic acid phenethyl ester (CAPE) on membrane potential and intracellular calcium in human endothelial cells. Eur. Rev. Med. Pharmacol. Sci..

[22] Tallini Y.N., Brekke J.F., Shui B., Doran R., Hwang S.M., Nakai J. (2007). Propagated endothelial Ca2+ waves and arteriolar dilation in vivo: measurements in Cx40BAC GCaMP2 transgenic mice. Circ. Res..

[23] Earley S., Brayden J.E. (2015). Transient receptor potential channels in the vasculature. Physiol. Rev..

[24] Hong K.S., Lee M.G. (2020). Endothelial Ca(2+) signaling-dependent vasodilation through transient receptor potential channels. KOREAN J. PHYSIOL. PHARMACOL..

[25] Poblete I.M., Orliac M.L., Briones R., Adler-Graschinsky E., Huidobro-Toro J.P. (2005). Anandamide elicits an acute release of nitric oxide through endothelial TRPV1 receptor activation in the rat arterial mesenteric bed. J. Physiol..

[26] Zhang D.X., Mendoza S.A., Bubolz A.H., Mizuno A., Ge Z.D., Li R. (2009). Transient receptor potential vanilloid type 4-deficient mice exhibit impaired endothelium-dependent relaxation induced by acetylcholine in vitro and in vivo. Hypertension.

[27] Yang D., Luo Z., Ma S., Wong W.T., Ma L., Zhong J. (2010). Activation of TRPV1 by dietary capsaicin improves endothelium-dependent vasorelaxation and prevents hypertension. Cell Metabol..

[28] Wen X., Peng Y., Zheng B., Yang S., Han J., Yu F. (2022). Silybin induces endothelium-dependent vasodilation via TRPV4 channels in mouse mesenteric arteries. Hypertens. Res..

[29] Seki T., Goto K., Kiyohara K., Kansui Y., Murakami N., Haga Y. (2017). Downregulation of endothelial transient receptor potential vanilloid type 4 channel and small-conductance of Ca2+-activated K+ channels underpins impaired endothelium-dependent hyperpolarization in hypertension. Hypertension.

[30] Zhang L., Lu W., Lu C., Guo Y., Chen X., Chen J. (2022). Beneficial effect of capsaicin via TRPV4/EDH signals on mesenteric arterioles of normal and colitis mice. J. Adv. Res..

[31] Caterina M.J., Schumacher M.A., Tominaga M., Rosen T.A., Levine J.D., Julius D. (1997). The capsaicin receptor: a heat-activated ion channel in the pain pathway. Nature.

[32] Cheng X.L., Ruan Y.L., Dai J.Y., Fan H.Z., Ling J.Y., Chen J. (2024). 8-shogaol derived from dietary ginger alleviated acute and inflammatory pain by targeting TRPV1. Phytomedicine.

[33] Dedov V.N., Tran V.H., Duke C.C., Connor M., Christie M.J., Mandadi S. (2002). Gingerols: a novel class of vanilloid receptor (VR1) agonists. Br. J. Pharmacol..

[34] Mou A., Sun F., Tong D., Wang L., Lu Z., Cao T. (2024). Dietary apigenin ameliorates obesity-related hypertension through TRPV4-dependent vasorelaxation and TRPV4-independent adiponectin secretion. Biochim. Biophys. Acta, Mol. Basis Dis..

[35] Peixoto-Neves D., Wang Q., Leal-Cardoso J.H., Rossoni L.V., Jaggar J.H. (2015). Eugenol dilates mesenteric arteries and reduces systemic BP by activating endothelial cell TRPV4 channels. Br. J. Pharmacol..

[36] Edelstein A.D., Tsuchida M.A., Amodaj N., Pinkard H., Vale R.D., Stuurman N. (2014). Advanced methods of microscope control using μManager software. JBM.

[37] Hernández H., Parra A., Tobar N., Molina J., Kallens V., Hidalgo M. (2018). Insights into the HyPer biosensor as molecular tool for monitoring cellular antioxidant capacity. Redox Biol..

[38] Burgos P., Zúñiga R., Domínguez P., Delgado-López F., Plant L.D., Zúñiga L. (2014). Differential expression of two-pore domain potassium channels in rat cerebellar granule neurons. Biochem. Biophys. Res. Commun..

[39] Yan L., Wang J., Pan M., Qiu Q., Huang W., Qian H. (2016). Synthesis of analogues of BCTC incorporating a pyrrolidinyl linker and biological evaluation as transient receptor potential vanilloid 1 antagonists. Chem. Biol. Drug Des..

[40] Weil A., Moore S.E., Waite N.J., Randall A., Gunthorpe M.J. (2005). Conservation of functional and pharmacological properties in the distantly related temperature sensors TRVP1 and TRPM8. Mol. Pharmacol..

[41] Lucius A., Khajavi N., Reinach P.S., Köhrle J., Dhandapani P., Huimann P. (2016). 3-Iodothyronamine increases transient receptor potential melastatin channel 8 (TRPM8) activity in immortalized human corneal epithelial cells. Cell. Signal..

[42] Yang F., Xiao X., Cheng W., Yang W., Yu P., Song Z. (2015). Structural mechanism underlying capsaicin binding and activation of the TRPV1 ion channel. Nat. Chem. Biol..

[43] Nieto-Posadas A., Picazo-Juárez G., Llorente I., Jara-Oseguera A., Morales-Lázaro S., Escalante-Alcalde D. (2011). Lysophosphatidic acid directly activates TRPV1 through a C-terminal binding site. Nat. Chem. Biol..

[44] Morales-Lazaro S.L., Llorente I., Sierra-Ramirez F., Lopez-Romero A.E., Ortiz-Renteria M., Serrano-Flores B. (2016). Inhibition of TRPV1 channels by a naturally occurring omega-9 fatty acid reduces pain and itch. Nat. Commun..

[45] Paracatu L.C., Faria C.M., Quinello C., Renno C., Palmeira P., Zeraik M.L. (2014). Caffeic Acid phenethyl ester: consequences of its hydrophobicity in the oxidative functions and cytokine release by leukocytes. Evid Based Complement Alternat Med.

[46] Wang T., Chen L., Wu W., Long Y., Wang R. (2008). Potential cytoprotection: antioxidant defence by caffeic acid phenethyl ester against free radical-induced damage of lipids, DNA, and proteins. Can. J. Physiol. Pharmacol..

[47] Shao B., Mao L., Tang M., Yan Z.-Y., Shao J., Huang C.-H. (2021). Caffeic acid phenyl ester (CAPE) protects against iron-mediated cellular DNA damage through its strong iron-binding ability and high lipophilicity. Antioxidants.

[48] Salazar H., Llorente I., Jara-Oseguera A., García-Villegas R., Munari M., Gordon S.E. (2008). A single N-terminal cysteine in TRPV1 determines activation by pungent compounds from onion and garlic. Nat. Neurosci..

[49] Markvicheva K.N., Bogdanova E.A., Staroverov D.B., Lukyanov S., Belousov V.V. (2019). Imaging of intracellular hydrogen peroxide production with HyPer upon stimulation of HeLa cells with EGF. Methods Mol. Biol..

[50] Bilan D.S., Pase L., Joosen L., Gorokhovatsky A.Y., Ermakova Y.G., Gadella T.W.J. (2013). HyPer-3: a genetically encoded H_2_O_2_ probe with improved performance for ratiometric and fluorescence lifetime imaging. ACS Chem. Biol..

[51] Zhuravlev A., Ezeriņa D., Ivanova J., Guriev N., Pugovkina N., Shatrova A. (2024). HyPer as a tool to determine the reductive activity in cellular compartments. Redox Biol..

[52] Revazian A., Nesterenko A., Ezeriņa D., Luo T., Vertommen D., Gibhardt C.S. (2024). Thermal Proteome Profiling reveals rapid proteomic responses to redox changes in specific cellular compartments. bioRxiv.

[53] Haendeler J., Hoffmann J., Tischler V., Berk B.C., Zeiher A.M., Dimmeler S. (2002). Redox regulatory and anti-apoptotic functions of thioredoxin depend on S-nitrosylation at cysteine 69. Nat. Cell Biol..

[54] Yang H., Zhao N., Lv L., Yan X., Hu S., Xu T. (2017). Functional research and molecular mechanism of Kainic acid-induced denitrosylation of thioredoxin-1 in rat hippocampus. Neurochem. Int..

[55] Hao Y., Yang W., Ren J., Hall Q., Zhang Y., Kaplan J.M. (2018). Thioredoxin shapes the C. elegans sensory response to Pseudomonas produced nitric oxide. Elife.

[56] Sheth S.S., Castellani L.W., Chari S., Wagg C., Thipphavong C.K., Bodnar J.S. (2005). Thioredoxin-interacting protein deficiency disrupts the fasting-feeding metabolic transition. J. Lipid Res..

[57] Bodnar J.S., Chatterjee A., Castellani L.W., Ross D.A., Ohmen J., Cavalcoli J. (2002). Positional cloning of the combined hyperlipidemia gene Hyplip1. Nat. Genet..

[58] Waldhart A.N., Dykstra H., Peck A.S., Boguslawski E.A., Madaj Z.B., Wen J. (2017). Phosphorylation of TXNIP by AKT mediates acute influx of glucose in response to insulin. Cell Rep..

[59] Guo Y., Lu C., Zhang L., Wan H., Jiang E., Chen Y. (2021). Nutrient-induced hyperosmosis evokes vasorelaxation via TRPV1 channel-mediated, endothelium-dependent, hyperpolarisation in healthy and colitis mice. Br. J. Pharmacol..

[60] Sonkusare S.K., Bonev A.D., Ledoux J., Liedtke W., Kotlikoff M.I., Heppner T.J. (2012). Elementary Ca2+ signals through endothelial TRPV4 channels regulate vascular function. Science.

[61] Ledoux J., Taylor M.S., Bonev A.D., Hannah R.M., Solodushko V., Shui B. (2008). Functional architecture of inositol 1,4,5-trisphosphate signaling in restricted spaces of myoendothelial projections. Proc. Natl. Acad. Sci. U. S. A..

[62] Zhang L., Rong S., Dong H. (2025). Functional heterogeneity of endothelium-dependent vasorelaxation in different order branches of mesenteric artery in female/male mice. Microvasc. Res..

[63] Zuccolo E., Kheder D.A., Lim D., Perna A., Nezza F.D., Botta L. (2019). Glutamate triggers intracellular Ca(2+) oscillations and nitric oxide release by inducing NAADP- and InsP(3) -dependent Ca(2+) release in mouse brain endothelial cells. J. Cell. Physiol..

[64] Iraz M., Fadillioglu E., Tasdemir S., Erdogan S. (2005). Role of vagal activity on bradicardic and hypotensive effects of caffeic acid phenethyl ester (CAPE). Cardiovasc. Toxicol..

[65] Long Y., Han M., Chen J., Tian X.Z., Chen Q., Wang R. (2009). The vasorelaxant effect of caffeic acid phenethyl ester on porcine coronary artery ring segments. Vasc. Pharmacol..

[66] Gun A., Ozer M.K., Bilgic S., Kocaman N., Ozan G. (2016). Effect of caffeic acid phenethyl ester on vascular damage caused by consumption of high fructose corn syrup in rats. Oxid. Med. Cell. Longev..

[67] DelloStritto D.J., Connell P.J., Dick G.M., Fancher I.S., Klarich B., Fahmy J.N. (2016). Differential regulation of TRPV1 channels by H2O2: implications for diabetic microvascular dysfunction. Basic Res. Cardiol..

[68] Vriens J., Appendino G., Nilius B. (2009). Pharmacology of vanilloid transient receptor potential cation channels. Mol. Pharmacol..

[69] Schneider J.C., El Kebir D., Chéreau C., Lanone S., Huang X.L., De Buys Roessingh A.S. (2003). Involvement of Ca2+/calmodulin-dependent protein kinase II in endothelial NO production and endothelium-dependent relaxation. Am. J. Physiol. Heart Circ. Physiol..

[70] Jin S.W., Choi C.Y., Hwang Y.P., Kim H.G., Kim S.J., Chung Y.C. (2016). Betulinic acid increases eNOS phosphorylation and NO synthesis via the calcium-signaling pathway. J. Agric. Food Chem..

[71] Wong B.J., Fieger S.M. (2010). Transient receptor potential vanilloid type-1 (TRPV-1) channels contribute to cutaneous thermal hyperaemia in humans. J. Physiol..

[72] Agarwal S.C., Allen J., Murray A., Purcell I.F. (2010). Comparative reproducibility of dermal microvascular blood flow changes in response to acetylcholine iontophoresis, hyperthermia and reactive hyperaemia. Physiol. Meas..

[73] Price D.T., Vita J.A., Keaney J.F. (2000). Redox control of vascular nitric oxide bioavailability. Antioxidants Redox Signal..

[74] Natarajan K., Singh S., Burke T.R., Grunberger D., Aggarwal B.B. (1996). Caffeic acid phenethyl ester is a potent and specific inhibitor of activation of nuclear transcription factor NF-kappa B. Proc. Natl. Acad. Sci. U. S. A..

[75] Fitzpatrick L.R., Wang J., Le T. (2001). Caffeic acid phenethyl ester, an inhibitor of nuclear factor-kappaB, attenuates bacterial peptidoglycan polysaccharide-induced colitis in rats. J. Pharmacol. Exp. Therapeut..

[76] Al-Ghamdi A.Y. (2024). Caffeic acid phenethyl ester attenuates Enterococcus faecalis infection in vivo: antioxidants and NF-κB have a protective role against stomach damage. J Med Life.

[77] Hwang S., Jo M., Hong J.E., Kim W.S., Kang D.H., Yoo S.H. (2024). Caffeic acid phenethyl ester administration reduces enterotoxigenic Bacteroides fragilis-induced colitis and tumorigenesis. Toxins.

[78] Chen L., Sun B.B., Wang T., Wang X., Li J.Q., Wang H.X. (2010). Cigarette smoke enhances {beta}-defensin 2 expression in rat airways via nuclear factor-{kappa}B activation. Eur. Respir. J..

[79] Ma Y., Zhang J.X., Liu Y.N., Ge A., Gu H., Zha W.J. (2016). Caffeic acid phenethyl ester alleviates asthma by regulating the airway microenvironment via the ROS-responsive MAPK/Akt pathway. Free Radic. Biol. Med..

[80] Pandurangan A.K., Mohebali N., Hasanpourghadi M., Esa N.M. (2022). Caffeic acid phenethyl ester attenuates dextran sulfate sodium-induced ulcerative colitis through modulation of NF-κB and cell adhesion molecules. Appl. Biochem. Biotechnol..

[81] Lakhdari O., Cultrone A., Tap J., Gloux K., Bernard F., Ehrlich S.D. (2010). Functional metagenomics: a high throughput screening method to decipher microbiota-driven NF-κB modulation in the human gut. PLoS One.

[82] Wei X., Dai J., Liu R., Wan G., Gu S., Du Y. (2024). S/O/W emulsion with CAPE ameliorates DSS-induced colitis by regulating NF-κB pathway, gut microbiota and fecal metabolome in C57bl/6 mice. Nutrients.

[83] Kim C.S., Kawada T., Kim B.S., Han I.S., Choe S.Y., Kurata T. (2003). Capsaicin exhibits anti-inflammatory property by inhibiting IkB-a degradation in LPS-stimulated peritoneal macrophages. Cell. Signal..

[84] Ghorbanpour A., Salari S., Baluchnejadmojarad T., Roghani M. (2023). Capsaicin protects against septic acute liver injury by attenuation of apoptosis and mitochondrial dysfunction. Heliyon.

[85] Becatti M., Prignano F., Fiorillo C., Pescitelli L., Nassi P., Lotti T. (2010). The involvement of Smac/DIABLO, p53, NF-kB, and MAPK pathways in apoptosis of keratinocytes from perilesional vitiligo skin: protective effects of curcumin and capsaicin. Antioxidants Redox Signal..

[86] Saha K., Sarkar D., Khan U., Karmakar B.C., Paul S., Mukhopadhyay A.K. (2022). Capsaicin inhibits inflammation and gastric damage during H pylori infection by targeting NF-kB-miRNA Axis. Pathogens.

[87] Gardana C., Simonetti P., Berti C., Pietta P. (2007). Evaluation of propolis polyphenols absorption in humans by liquid chromatography/tandem mass spectrometry. Rapid Commun. Mass Spectrom..

[88] Tolba M.F., Azab S.S., Khalifa A.E., Abdel-Rahman S.Z., Abdel-Naim A.B. (2013). Caffeic acid phenethyl ester, a promising component of propolis with a plethora of biological activities: a review on its anti-inflammatory, neuroprotective, hepatoprotective, and cardioprotective effects. IUBMB Life.

[89] Celli N., Dragani L.K., Murzilli S., Pagliani T., Poggi A. (2007). In vitro and in vivo stability of caffeic acid phenethyl ester, a bioactive compound of propolis. J. Agric. Food Chem..

[90] Maria Parro Y., de Mendonça Guimarães D., Sampaio Muller H., Barbosa Coelho E., Aparecida Berretta A., Aparecida de Lima J. (2022). Efficacy of a 0.5% propolis -0.9% pomegranate buccal spray treatment compared with 2% miconazole gel for denture stomatitis treatment in elderly patients: a randomized clinical trial. J. Dent..

[91] Kapare H.S., Lohidasan S., Sinnathambi A., Mahadik K. (2021). Formulation development of folic acid conjugated PLGA nanoparticles for improved cytotoxicity of caffeic acid phenethyl ester. Pharm. Nanotechnol..

