# Supplementary material for: The antioxidant property of CAPE depends on TRPV1 channel activation in microvascular endothelial cells
Source: Redox Biol. 2025 Jan 20;80:103507. doi: 10.1016/j.redox.2025.103507 (PMC11794178; doi:10.1016/j.redox.2025.103507)
Supplement: Multimedia component 1 [file mmc1.pptx]

## Slide 1
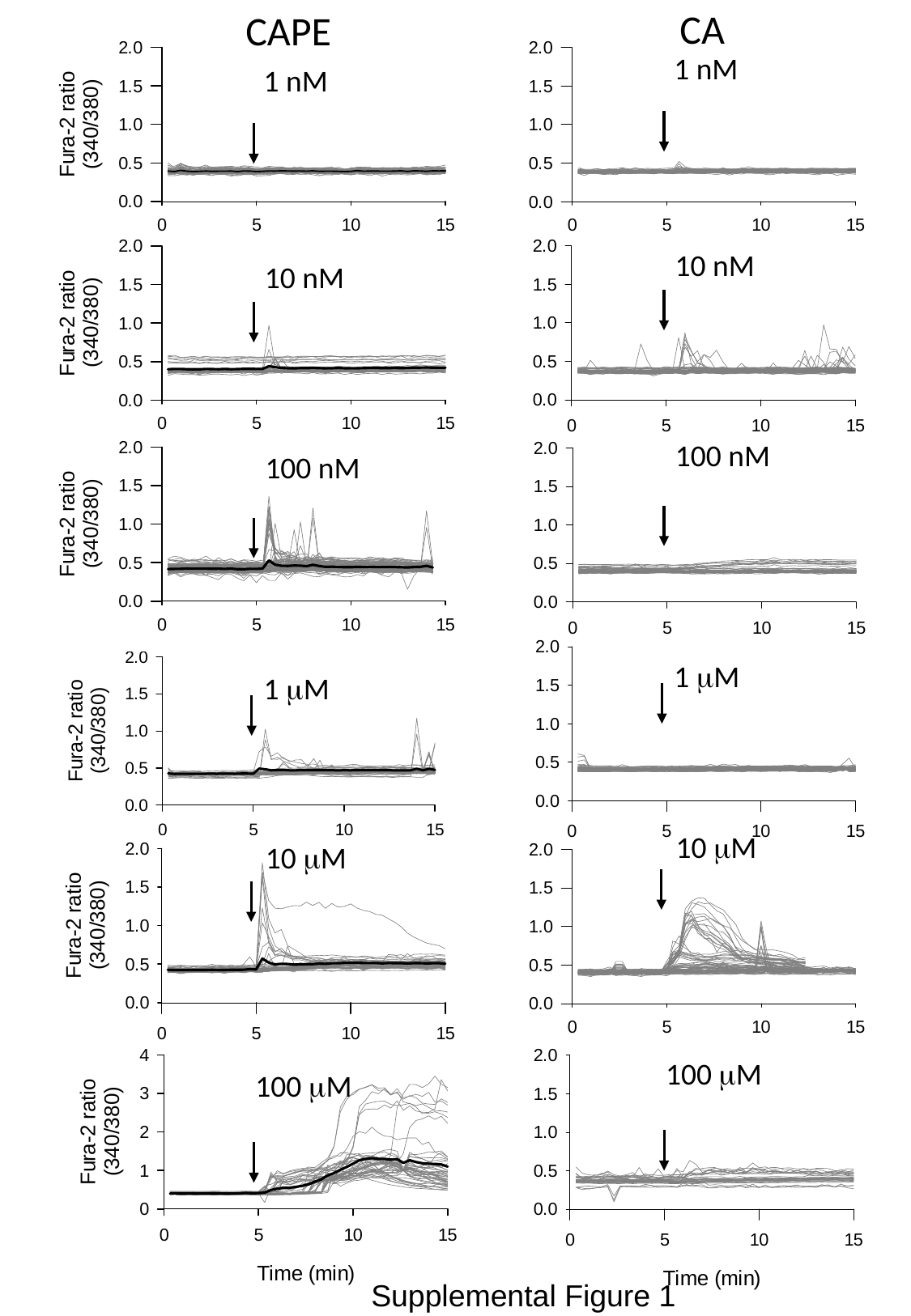

CA
CAPE
1 nM
1 nM
10 nM
10 nM
100 nM
100 nM
1 mM
1 mM
10 mM
10 mM
100 mM
100 mM
Supplemental Figure 1

## Slide 2
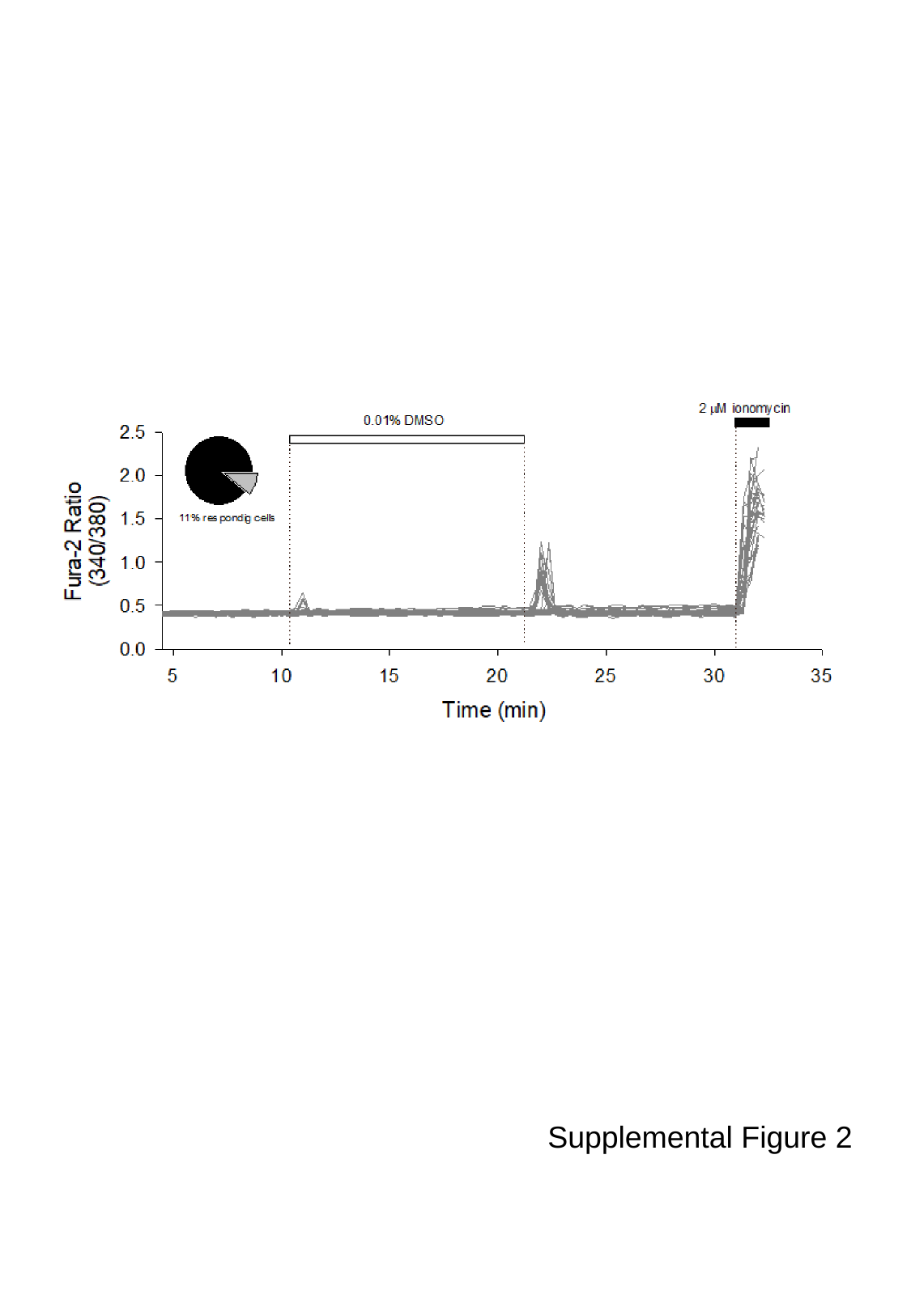

Supplemental Figure 2

## Slide 3
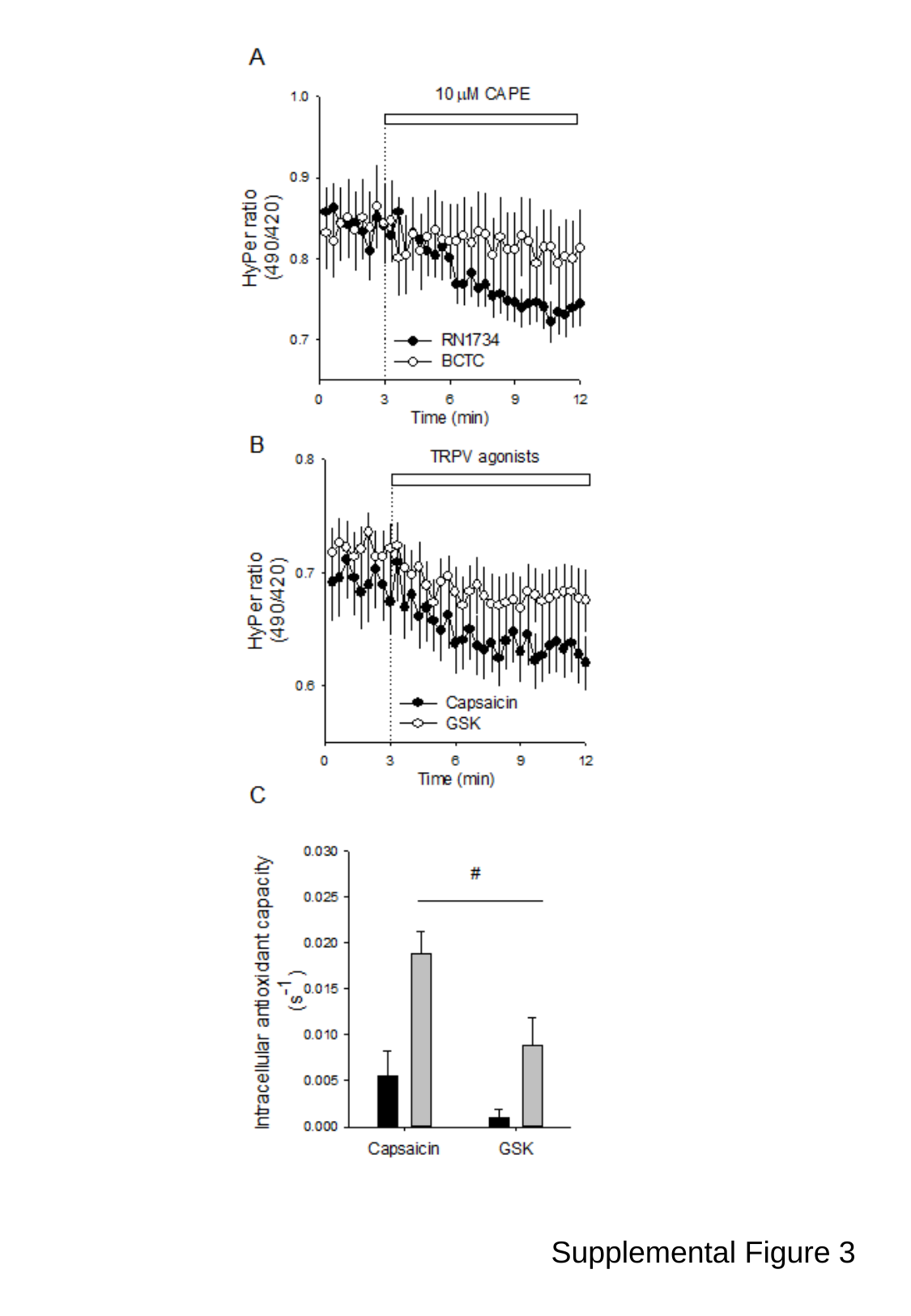

Supplemental Figure 3
